# Synthesis, Surface Activity, Emulsifiability and Bactericidal Performance of Zwitterionic Tetrameric Surfactants

**DOI:** 10.3390/molecules29184286

**Published:** 2024-09-10

**Authors:** Xin Wei, Jie Li, Xiangfei Geng, Di Niu, Zhenjie Wei, Chenxu Wang, Ziqi Sun, Yangchun Xie

**Affiliations:** 1 College of Chemistry and Chemical Engineering, Northeast Petroleum University, Daqing 163318, China; 18546687837@163.com (X.W.); 18847138387@163.com (Z.W.); 13204597627@163.com (C.W.); 13115589952@163.com (Z.S.); 18003691285@163.com (Y.X.); 2 Key Laboratory of Oilfield Chemicals, China National Petroleum Corporation, Beijing 100083, China; 3 PetroChina Research Institute of Petroleum Exploration & Development, Beijing 100083, China; 4 Zhonghao Guangming Research & Design Institute of Chemical Industry Co., Ltd., Dalian 116031, China; niudi6663@163.com

**Keywords:** zwitterionic tetrameric surfactants, critical aggregation concentration, antibacterial activity, emulsification

## Abstract

In this paper, a series of tetrameric surfactants (4C_n_SAZs, *n* = 12, 14, 16) endowed with zwitterionic characteristic were synthesized by a simple and convenient method and their structures were characterized by FT-IR, ^1^H NMR and elemental analysis. Their physicochemical properties were studied using the Wilhelmy plate method, fluorescence spectra and dynamic light scattering technique. 4C_n_SAZs have higher surface activities and tend to adsorb at the air/water surface rather than self-assembling in aqueous solution. The thermodynamic parameters obtained from surface tension measurements show that both processes of adsorption and micellization of 4C_n_SAZs are spontaneous and that the micellization processes of 4C_n_SAZs are entropy-driven processes. Both adsorption and micellization of 4C_n_SAZs are inclined to occur with the increase of alkyl chain length or temperature. For 4C_12_SAZs, there are only small-size aggregates (micelles), while the large aggregates (vesicles) are observed at the alkyl length of 4C_n_SAZs of 14 or 16. This shows that the alkyl chain length for oligomeric surfactants has a greater sensitivity for aggregate growth. The aggregate morphologies obtained from the calculated values of critical packing parameter (*p*) for 4C_14_SAZs and 4C_16_SAZs can be supported by the DLS measurement results. The test results obtained by the separation-water-time method show that 4C_n_SAZs have good emulsification performance and that the prepared emulsions appear to exit in the form of multiple emulsions. In addition, 4C_n_SAZs have good antibacterial activities against *Escherichia coli* (*E. coli*) and Staphylococcus aureus (*S. aureus*). The present study reveals the unique behavior of a zwitterionic tetrameric surfactant and may give new insights into molecular design and synthesis of a high degree of surfactants with different structure characteristics for potential application in various industrial fields.

## 1. Introduction

Zwitterionic surfactants are amphiphilic molecules, in which their two polar groups (basic and acidic groups) that exhibit amphoteric characters are connected through a spacer group [1,2]. Zwitterionic surfactants contain no counterions, and conversely, molecules bear a balance of positively and negatively charged functional groups in a similar way as many amino acids [3,4]. They have a lot of superior properties such as good water solubility, salt resistance, high-temperature resistance, and good synergistic effect, as compared with other ionic surfactants [5,6]. These inherent features make them gain considerable attention, both in the academic field and industrial applications [7]. At present, there are many different zwitterionic surfactants synthesized and the demand for this kind of surfactant is increasing each year [8].

Gemini surfactants, also called dimeric surfactants, consist of two polar head groups and two hydrophobic groups covalently connected by spacer groups [9,10], and were known as a new generation of surfactants in the 1990s [4]. Because the kind of surfactants exhibits unique physicochemical properties such as lower critical aggregation concentration (CAC), higher efficiency to reduce surface tension, and greater wettability and emulsifiability as compared with the conventional monomeric surfactants, they have become one of the research focuses in the colloid and interface chemistry [11,12,13,14]. 

Over the past few decades, more research in the area has focused on the synthesis of different structures of gemini surfactants such as heteroatoms [15,16] or zwitterionic structures [17,18] and their physicochemical properties. So far, a more systematic research is the structure-effective relationship of linear gemini surfactants because of their simple synthesis methods. For zwitterionic gemini surfactants containing two different charged ionic or nonionic groups as hydrophilic moieties, there is a smaller electrostatic repulsion between the hydrophilic head groups of the same molecule or different molecules, and this helps improve a compact arrangement at the interface and greatly reduces surface tensions [19,20]. The existence of zwitterions in the same molecule makes them have a unique aggregation morphology and unique rheology in aqueous solution, which makes them increase more potential applications. Zhou [21] and Cheng [22] independently reviewed the developments of zwitterionic gemini surfactants in sequence until 2019. Xu et al. [23] reported the synthesis of a novel trimeric octadecyl zwitterionic surfactant (TOCC), and its three branched chains contain six quaternary ammonium atoms and three carboxylate groups. The unique structure of TOCC enriches the variety of trimeric surfactants and results in the electropositivity of TOCC. Park et al. [24] synthesized an eco-friendly phospholipid-based zwitterionic gemini surfactant (CDP-W) with coconut oil source, and CDP-W molecules have two quaternary ammonium atoms and a phosphate group. CDP-W with superior antiseptic and antiviral performances can replace cocamidopropyl betaine in the formulation of cosmetics, personal products and household products.

To meet the increasing needs of industry and life in lots of aspects for surfactants and excellent properties of dimeric surfactants, researchers are stimulated to synthesize surfactants with three or more hydrophobic and hydrophilic groups and study their basic physicochemical properties. This kind of surfactants is again referred to as oligomeric surfactants, such as trimeric or tetrameric surfactants. According to spacer structures, oligomeric surfactants may be divided into three main types, line-type, star-type and ring-type, while the reports on the ring-type are relatively few [25,26]. Most of the pioneering works on synthesis, self-assembly and rheological properties of linear oligomeric surfactants, referred as as the natural extension of Gemini surfactants in one dimension, have been done by Zana et al. [27,28,29]. There are more spacer groups in a molecule, which makes the synergistic effect between multiple alkyl chains stronger and the electrostatic repulsion among charged headgroups smaller. Thus, as compared with single-chain and gemini surfactants, the linear oligomeric surfactants are more inclined to adsorb at the interface or form abundant aggregate structures in water solution. The star-shaped oligomeric surfactants display symmetric or dissymmetric dendritic configurations and branched three-dimensional structures. Their spacer groups derive from a central moiety such as an atom or a group of atoms, and the polar head groups are covalently linked with hydrophobic chains or planar aromatic groups [30]. The special structures of such surfactants may overcome the rigidity of the spacer groups, decrease intermolecular electrostatic repulsion, and enhance the hydrophobic association among alkyl chains of the same or different molecules. 

Menger et al. [31] synthesized three series of star-shaped cationic oligomeric surfactants with pentaerythritol (PE), dipentaerythritol (DPE), and adamantane (AD) as a core, and only solubility and aggregate behavior were studied. Marangoni et al. [32,33,34] also reported the synthesis of five series of cationic, three series of anionic, and zwitterionic gemini surfactants from pentaerythritol. In addition, a family of three-headed five-tailed oligomeric surfactants were also synthesized with tripentaerythritol as a backbone. The synthesis routes of all the above surfactants are complex, and some of the reagents and catalysts are unstable and toxic. Asadov et al. [35] synthesized a nonionic oligomeric surfactant by oxypropylation reaction via pentaerythritol and propylene oxide with an alkali catalyst, and this product has the inhibition ability of gasoline evaporation, and good industrial prospect because of relatively low cost and availability of raw materials. Abdel-Raouf [36] carried out etherification by polyethylene glycol and pentaerythritol, and then the intermediate reacted with fatty acid and obtained biodegradable tetrameric star-shaped surfactants. These surfactants may act as oil spill dispersants and have a characteristic of mature production technology.

In this study, a series of zwitterionic tetrameric surfactants (4C_n_SAZs) were prepared just by sulfating four hydroxyl groups of 4C_n_tetraQs synthesized in our previous work [37]. The reaction routes include three cheap, mild reactions, and they are etherification after ring opening for epichlorohydrin, quaterization for trialkylamine, and sulfation of hydroxyl groups. All these unit operations are mature technologies and have the advantages of wide raw material sources, convenient treatment, and simple production. The process combination of three common cheap reactions may make the difficult synthesis of the oligomeric surfactants with complex structures become easier. The physicochemical properties of 4C_n_SAZs were studied by the Wilhelmy plate method, fluorescence spectra and dynamic light scattering technique. The emulsification properties of 4C_n_SAZs were investigated by separation-water-time method and micrography. The antibacterial activities of 4C_n_SAZs against *E. coli* and *S. aureus* were performed by plate count method.

## 2. Results and Discussion

### 2.1. Structural Characterization

**Figure 1:**
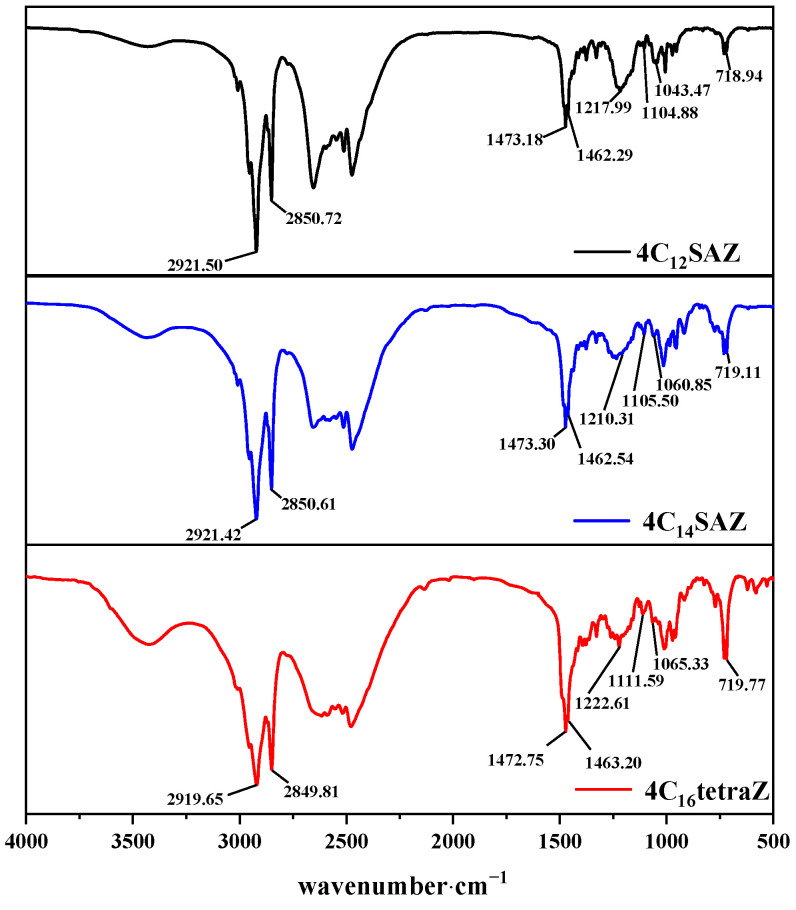
Infrared spectrum of zwitterionic tetrameric surfactants (4C_n_SAZs, *n* = 12, 14, 16).

**Figure 2:**
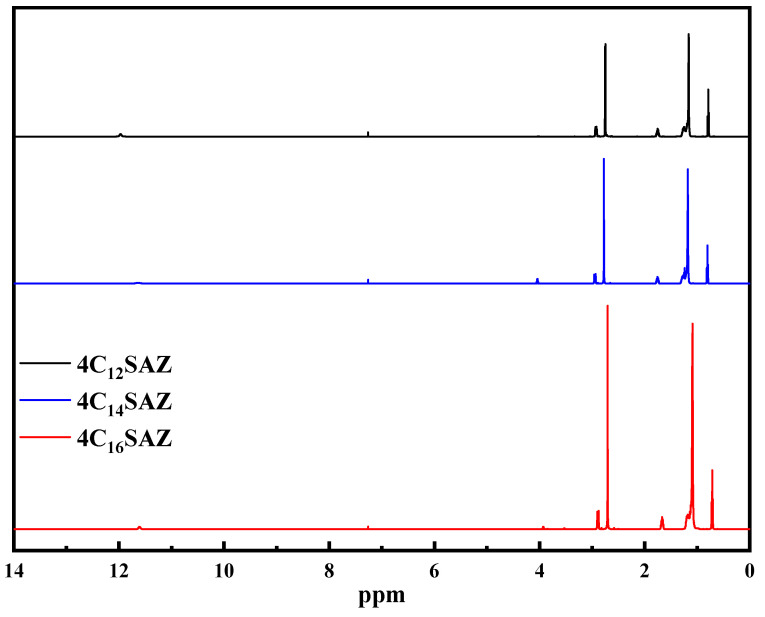
^1^H NMR spectra of zwitterionic tetrameric surfactants (4C_n_SAZs, *n* = 12, 14, 16).

**Figure 3:**
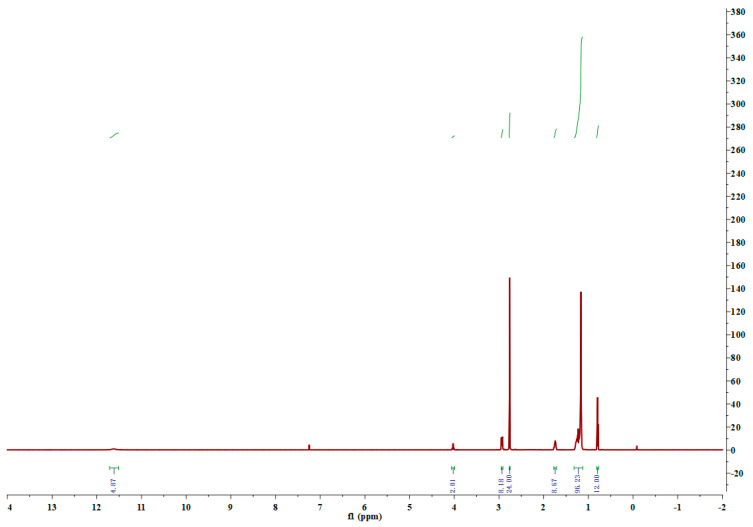
Integrated curve of 4C_14_SAZ nuclear magnetic resonance hydrogen spectrum.

The structures of 4C_n_SAZs were characterized by ^1^H NMR, FT-IR and elemental analysis. The characteristic results for FT-IR and ^1^H NMR are given in Figure 1, Figure 2 and Figure 3, in which the integration curve of ^1^H NMR for 4C_14_SAZ is shown in Figure 3 as an example. 

4C_12_SAZ: FT-IR (KBr), 2921.50, 2850.72 cm^−1^ (C–H stretching); 1473.18, 1462.29 cm^−1^ (C–H bending in-plane); 1217.99 cm^−1^ (–SO_3_– stretching); 1104.88 cm^−1^ (C–O–C stretching); 1043.47 cm^−1^ (C–N^+^ stretching); 718.94 cm^−1^ (–CH_2_– stretching). ^1^H NMR (600 MHz, CDCl_3_, ppm), δ: 0.79 (t, 12H, CH_3_–CH_2_); 1.16 (m, 80H, CH_3_–(CH_2_)_10_); 1.74 (s, 8H, CH_3_–(CH_2_)_10_–CH_2_); 2.75 (d, 24H, CH_3_–N^+^–CH_3_); 2.92 (m, 8H, CH_2_–N^+^); 3.94 (t, 4H, CH–O–SO_3_H); 4.02 (q, 16H, O–CH_2_); 11.97 (s, 4H, O–SO_3_–H). Anal. Calcd. for C_73_H_156_O_20_S_4_N_4_ %: C, 57.0; H, 10.22; N, 3.64. Found %: C, 56.88; H, 10.28; N, 3.59. 

4C_14_SAZ: FT-IR (KBr), 2921.42, 2850.61 cm^−1^ (C–H stretching); 1473.30, 1462.54 cm^−1^ (C–H bending in–plane); 1210.31 cm^−1^ (–SO_3_– stretching); 1105.50 cm^−1^(C–O–C stretching); 1060.85 cm^−1^ (C–N^+^ stretching); 719.11 cm^−1^ (–CH_2_– stretching). ^1^H NMR (600 MHz, CDCl_3_, ppm), δ: 0.81 (t, 12H, CH_3_–CH_2_); 1.18 (m, 96H, CH_3_–(CH_2_)_12_); 1.76 (m, 8H, CH_3_–(CH_2_)_12_–CH_2_); 2.78 (s, 24H, CH_3_–N^+^–CH_3_); 2.95 (m, 8H, CH_2_–N^+^); 3.96 (t, 4H, CH–O–SO_3_H); 4.05 (q, 16H, O–CH_2_); 11.65 (s, 4H, O–SO_3_–H). Anal. Calcd. for C_81_H_172_O_20_S_4_N_4_ %: C, 58.95; H, 10.5; N, 3.39. Found %: C, 58.81; H, 10.55; N, 3.35.

4C_16_SAZ: FT-IR (KBr), 2919.65, 2849.81 cm^−1^ (C–H stretching); 1472.75, 1463.20 cm^−1^ (C–H bending in–plane); 1222.61 cm^−1^ (–SO_3_– stretching); 1111.59 cm^−1^ (C–O–C stretching); 1065.33 cm^−1^ (C–N^+^ stretching); 719.77 cm^−1^ (–CH_2_– stretching). ^1^H NMR (600 MHz, CDCl_3_, ppm), δ: 0.81 (t, 12H, CH_3_–CH_2_); 1.18 (m, 96H, CH_3_–(CH_2_)_12_); 1.76 (m, 8H, CH_3_–(CH_2_)_12_–CH_2_); 2.78 (s, 24H, CH_3_–N^+^–CH_3_); 2.95 (m, 8H, CH_2_–N^+^); 3.96 (t, 4H, CH–O–SO_3_H); 4.05 (q, 16H, O–CH_2_); 11.65 (s, 4H, O–SO_3_–H). Anal. Calcd. for C_89_H_188_O_20_S_4_N_4_ %: C, 60.64; H, 10.75; N, 3.18. Found %: C, 60.56; H, 10.82; N, 3.11.

### 2.2. Surface Activity

**Figure 4:**
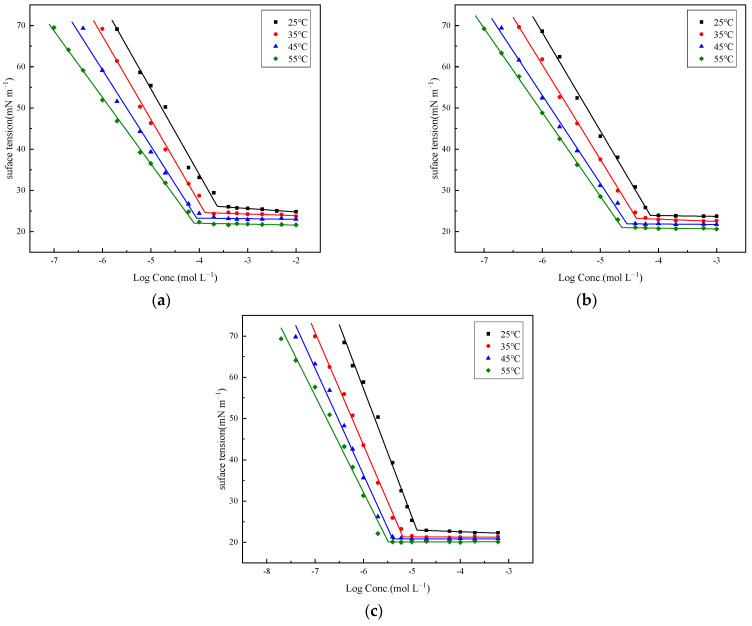
The curves of surface tension versus log concentration of 4C_n_SAZs at different temperature: (**a**) 4C_12_SAZ, (**b**) 4C_14_SAZ, (**c**) 4C_16_SAZ.

The surface tension measurement is a classical method of studying the critical aggregation concentration (CAC) of surfactants. The results of surface tension measurements for 4C_n_SAZs (*n* = 12, 14, 16) aqueous solutions were shown in Figure 4 at different temperatures, and the CAC and the surface tension at CAC (*γ*_CAC_) were given in Table 1.

As expected, zwitterionic tetrameric 4C_n_SAZs display remarkable physicochemical properties such as lower CAC and greater efficacy in lowering surface tension. For example, the CAC and the corresponding *γ*_CAC_ for 4C_12_SAZ at 25 °C are 0.236 mmol L^−1^ and 26.10 mN/m, respectively, are much lower than those of conventional ionic surfactants, for example, sodium dodecyl sulfonate (SDS, 7.25 mmol L^−1^, 34.8 mN/m) [38,39] and dodecanaminium bromide (DTAB, 15.7 mmol L^−1^, 38.9 mN/m) [40,41]. This implies that 4C_n_SAZs have excellent micelle-forming ability and the ability to reduce surface tension at low concentrations. 4C_n_SAZs have four alkyl chains closely connected in the single molecule, and the whole molecule is electrically neutral. This structure characteristic of 4C_n_SAZs can enhance the hydrophobic association among alkyl chains of the same or different molecules and decrease intermolecular electrostatic repulsion, which can result in more compact arrangement of 4C_n_SAZs molecules at the air-water interface. The longer the alkyl chain length of 4C_n_SAZs becomes, the lower the values of CAC and *γ*_CAC_ are [42]. These results may be accounted for by the fact that the hydrophobicity of the surfactant will increase with increasing length of the alkyl tail chain. The strong hydrophobic effect can destroy the water structure and increase the free energy of the system, which triggers the directed migration of surfactant molecules to the gas/liquid interface or aggregation into a micellar structure [43].

This behavior, in turn, results in the relaxation in the free energy of surfactant solution, consequently inducing a decrease in the CAC and *γ*_CAC_. In addition, as the temperature increases, the CAC and corresponding *γ*_CAC_ of 4C_n_SAZs decreases. The decrease of CAC may be due to the reduction of the water hydration degree of polar head groups that can help promote micellization. Moreover, the increase of solution temperature can break hydrogen bonding between water molecules and polar head groups and make head groups decrease their hydrophilicity [44]. The hydrophilicity reduction of head groups can promote surfactant molecules to escape from the water phase and closely adsorb at the air/water interface, resulting in lower surface tension. This is probably because the increasing temperature accelerates the thermal motion of surfactant molecules and weakens the electrostatic interaction between the head groups of surfactant molecules [45].

The surface adsorption parameters including maximum surface excess concentration (*Γ*_max_) and the area minimum (*A*_min_) were calculated with the Gibbs adsorption isotherm Equations (1) and (2) [46].

(1) $$\Gamma_{\max}=\frac{-1}{2.303\mathrm{nRT}}{\left(\frac{d\gamma }{d\log\;C}\right)}_{T}$$

(2) $$A_{\min}=\frac{1}{N_{A\;}\Gamma_{\max}}$$

where *R* is the gas constant (8.314 J mol^−1^ K^−1^); *T* is the thermodynamic temperature (K); *n* is a constant, and its value depends on the type of composition adsorbed at the air/water interface. The zwitterionic surfactant is an internal salt and the whole molecule is electrically neutral. Thus, the value of n is 1 [47,48]; (d*γ*/d*logC*) is the slope below the CAC in the surface-tension plots; *N*_A_ is the Avogadro’s number (6.02 × 10^23^). The *Γ*_max_ may indicate the amount of accumulated 4C_n_SAZs molecules in the interface, and *A*_min_ can reflect the tightness of the arrangement of surfaceactive molecules in the interface, with smaller values indicating a tighter arrangement.

The *A*_min_ values for 4C_n_SAZs are far smaller than those of the traditional single-chain surfactant, which indicate that 4C_n_SAZs can adsorb efficiently and pack tightly at the gas/water interface, forming a coherent interface film [49]. In turn, this also helps to explain that 4C_n_SAZs have an extremely low CAC. The neutralization of the same amount of positive and negative charges in the same molecule and the electrostatic attraction between the opposite charges of different molecules are also helpful for a tight package [8]. It is found that an increase in the hydrocarbon tail length from 12 to 16 may lead to an increase in *Γ*_max_ and a decrease in *A*_min_, in which these results are in accordance with the outcomes reported in other literature [50]. This is due to the increase of molecular structure complexity, steric hindrance of the alkyl chain, hydrophobic interaction force, and the decrease of hydrophilic-lipophilic equilibrium value (HLB). In addition, 4C_n_SAZs molecules were oriented horizontally, which may decrease the amount of the accumulated 4C_n_SAZs molecules at the interface [49].

Two significant parameters of investigated amphiphiles, i.e., the effectiveness of surface tension lowering (*π*_CAC_) and the adsorption efficiency (*pC*_20_), were determined from surface tension isotherms with the following Equations (3) and (4) [51].

(3) $$\pi_{\mathrm{CAC}}{=\gamma }_{0}-\gamma_{CAC}$$

(4) $${\mathrm{pC}}_{20}=-{\mathrm{logC}}_{20}$$

where *γ*_CAC_ and *γ*_0_ are the surface tension of surfactant solution at CAC and pure water, respectively; *C*_20_ is the concentration of surfactant required to produce a 20 mN/m reduction in surface tension.

*π*_CAC_ represents the effectiveness of amphiphilic molecules to maximal lower the surface tension of water. Therefore, *π*_CAC_ is used as a metric to assess the maximum reduction of surface tension [51,52]. Specifically, high values of *π*_CAC_ refer to the formation of a compact layer at the interface, whereas low values indicate loose packing of surfactant molecules at the interface [43]. The outcomes from Table 1 show that the *π*_CAC_ increases with the increase of hydrophobic chain length.

*pC*_20_ represents the efficiency of surfactants in reducing water surface tension. The larger the *pC*_20_, the higher its efficiency in reducing water surface tension, and the less amount required in practical applications. As can be seen from Table 1 that *pC*_20_ values for 4C_n_SAZs are two or more higher than those of the conventional ionic surfactants, such as SDS and CTAB, and this means that zwitterionic 4C_n_SAZs molecules are inclined to adsorption at the gas/liquid interface than those of the analogue ionic unimers.

### 2.3. Thermodynamics of Micellization/Thermodynamic Parameters

The thermodynamic parameters of the synthesized zwitterionic tetrameric surfactants at 25 °C, 35 °C, 45 °C, and 55 °C were calculated using the Gibbs Equations (5)–(8) [53,54], and are summarized in Table 2.

(5) $$\Delta G_{\mathrm{mic}}^{o}=\mathrm{RT}\ln X_{CAC}$$

(6) $$\Delta H_{mic}^{o}=-RT^{2}\frac{\partial lnX_{CAC}}{\partial T}$$

(7) $$\Delta S_{mic}^{o}=\frac{1}{T}\left(\Delta H_{m}^{o}-\Delta G_{m}^{o}\right)$$

(8) $$\Delta G_{ads}^{o}=\Delta G_{mic}^{o}-\frac{\pi_{CAC}}{\Gamma_{\max}}$$

where *R* is the gas constant, 8.314 J mol^−1^ K^−1^ and *T* is the absolute temperature; Δ*G*^o^_mic_ and Δ*G*^o^_ads_ are the standard Gibbs free energy changes of micellization and adsorption, respectively; Δ*H*^o^_mic_ is the standard enthalpy change of the micellization; Δ*S*^o^_mic_ is the standard entropy of micelle formation.

As can be seen from Table 2 that all values of Δ*G*^o^_mic_ and Δ*G*^o^_ads_ are negative, indicating that the micellization and adsorption of these surfactants are all spontaneous [55]. In this case, Δ*G*^o^_ads_ is more negative than the corresponding Δ*G*^o^_mic_, showing that adsorption is the primary process compared to micellization [53]. As temperature increases, the absolute values of Δ*G*^o^_mic_ and Δ*G*^o^_ads_ increase gradually. Thus, increasing temperature favors both micellization and adsorption of surfactant molecules. At the same time, the increase of alkyl chain length from 12 to 16 has the same impact as increasing temperature. The absolute values of Δ*G*^o^_ads_ are slightly bigger than those of Δ*G*^o^_mic_, showing that surfactant molecules have a big tendency to adsorb at the interface rather than form micelles [56].

All positive Δ*S*^o^_mic_ values indicate that the micellizations are the entropy-driven process. The increase of the disorder degree of the system may be attributable to the destruction/collapse of the “ice structure” consisting of water molecules around hydrophilic groups, whereas the aggregation process of surfactant molecules results in the decrease of the disorder degree of the system. In addition, the instability of the iceberg structure can also entice an increase in positive Δ*S*^o^_mic_. The increasing temperature will enhance the disorder degree of water molecules, which results in the increase of Δ*S*^o^_mic_ [57].

As shown in Table 2, Δ*H*^o^_mic_ are positive, indicating that both micellization and adsorption processes are controlled by the entropy change, and both processes are endothermic. The values of *−T*Δ*S*^o^_mic_ are far smaller than those of Δ*H*^o^_mic_, showing that micellization processes of surfactant molecules are controlled by entropy.

### 2.4. Micropolarity

As is well known that the micellization behavior of surfactants in an aqueous solution is obtained from a steady-state fluorescence spectrum with pyrene as a probe, in which the intensity ratio between the first and third vibrational peaks (I_1_/I_3_) in the fluorescence spectrum may be used to characterize the polarity of the probe’s microenvironment. The decrease in the I_1_/I_3_ ratio means that pyrene molecules are migrating from a polar region to a nonpolar region (aggregates).

**Figure 5:**
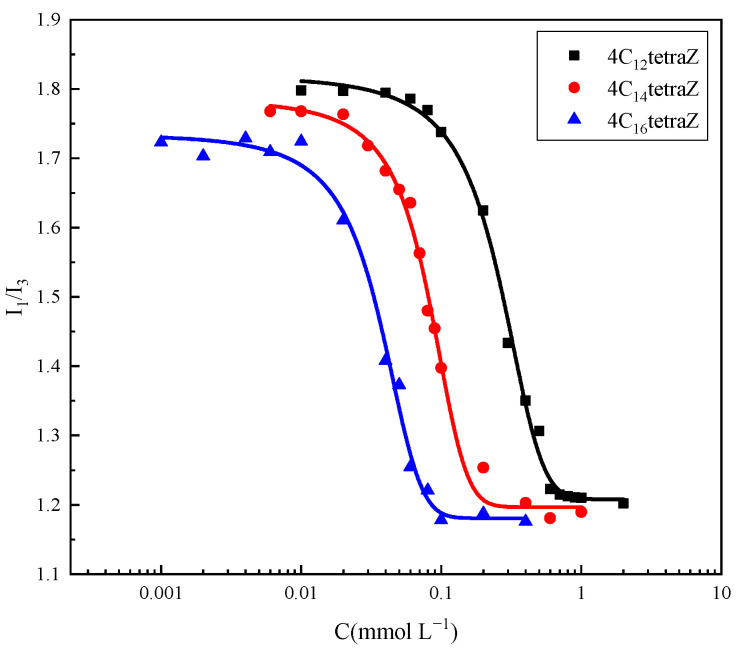
Variation of the pyrene fluorescence intensity ratio (I_1_/I_3_) vs. 4C_n_SAZs concentration at 25 °C.

Figure 5 shows variations of the pyrene polarity ratio (I_1_/I_3_) vs. 4C_n_SAZs concentration at 25 °C. As can be seen in Figure 5, the I_1_/I_3_ ratio changes sharply at the concentrations around the CAC, showing that a small number of aggregates begin to form and solubilize pyrene in the inner hydrophobic region of aggregates [57]. The minimum ratio of I_1_/I_3_ decreases with the increase of alkyl chain length, suggesting that the strong hydrophobic interaction among longer alkyl tails can make the palisade layer arrange more tightly and result in a reduction in polarity [55].

### 2.5. Critical Arrangement Parameters and Aggregate Morphology

The critical packing parameter (*P*) [55] is used to better reflect the geometric morphology of aggregates by the association of 4C_n_SAZs molecules in water, and may be calculated by the Equations (9)–(12) [58,59].

(9) $${P=V}_{\mathrm{hydrophobic}}/{(a}_{0}l_{0})$$

(10) $$V=(27.4+26.9m)\times 10^{-3}\;{\mathrm{nm}}^{3}$$

(11) $$l_{0}=(0.15+0.1265m)\;\mathrm{nm}$$

(12) $$R=\frac{4V}{{\;A}_{\min}}$$

where, *a*_0_ is the average parking area of the polar head group in a surfactant molecule. *V*_hydrophobic_ and *l*_0_ are the volume and length of an alkyl chain, respectively. Moreover, *m* is the number of carbon atoms in an alkyl chain.

The shape and geometry of the aggregates can be predicted according to the *p* values as shown in Table 3. According to the theory of critical packing parameter, when *p* values range from 0 to 0.33, the spherical micelles are inclined to form in the surfactant solution. The *p* values are from 0.33 to 0.5, for nonspherical micelles. It is 0.5 to 1, for bilayers or vesicles. As can be seen from Table 3, the *p* value of 4C_12_SAZ is 0.46, and this means that 4C_12_SAZ molecules may form nonspherical micelles in an aqueous solution. However, *p* values for 4C_14_SAZ and 4C_16_SAZ are above 0.5, showing that such surfactants with longer alkyl chains may form vesicle structures and this also is in accordance with the DLS results. The formation of bigger aggregates when *n* ≥ 14 may be caused by the stronger hydrophobicity imparted by the four longer hydrocarbon chains [42]. 

**Figure 6:**
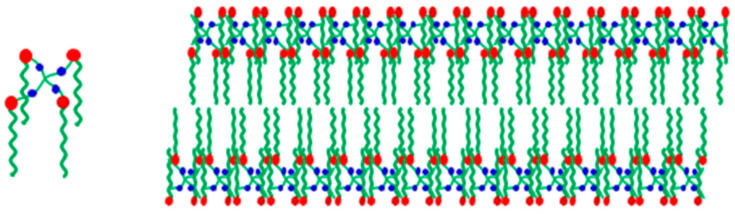
The possible aggregate model of 4C_14_SAZ and 4C_16_SAZ in the aqueous solution.

To study the arrangement of the alkyl chains of surfactant molecules in the vesicular region, the radius values of aggregates can be calculated by Tanford’s equation. As can be seen from Table 3, the *R* values are above twice the length of the hydrophobic chain fully extended, which means that the bilayer thickness of vesicles for 4C_14_SAZ and 4C_16_SAZ is above twice the hydrophobic chain length [58]. The alkyl chains of tetrameric surfactant molecules forming vesicle structures may present a face-to-face attitude and parallel arrangement. The possible aggregate model is given in Figure 6.

### 2.6. DLS

The DLS is one of the strongest tools used to determine the size distribution and speculate the shapes of various aggregates formed in the aqueous solution.

**Figure 7:**
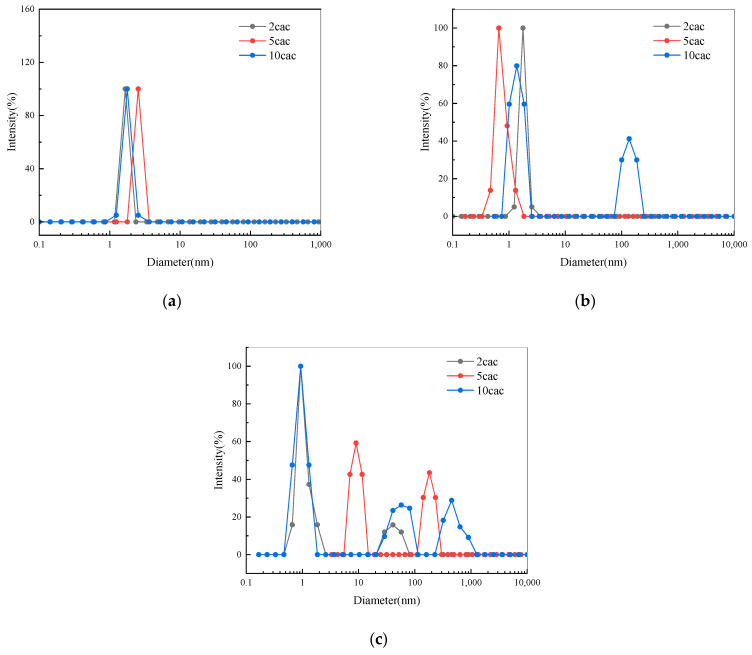
The aggregate hydrodynamic radius (*R_h_*) of 4C_n_SAZs: (**a**) 4C_12_SAZ, (**b**) 4C_14_SAZ, (**c**) 4C_16_SAZ.

As shown in Figure 7, when 4C_n_SAZs (*n* = 12, 14) concentration is 2 CAC, there are the small spherical micelles observed in all 4C_n_SAZs solutions. For 4C_12_SAZ, when its concentration ranges from 2 CAC to 10 CAC, there are only small size aggregates (1.6 nm) observed. These results are not in accordance with the results from the *p* value calculated, which may be due to the fact that the calculated value of average parking area (*a*_0_) of polar head groups of 4C_12_SAZ is smaller than its practical value. For 4C_14_SAZ, when its concentration is 10 CAC, the big aggregates (100–185 nm) may be observed. While the alkyl length increases to 16, aggregates of both small sizes and large ones can be observed at each concentration investigated, and the latter usually is assigned to the vesicle. The above results suggest that the alkyl chain length has a greater sensitivity for aggregate growth than the sizes of concentration [60]. These test results are in accordance with the hydrodynamic sizes of aggregates of gemini (G-12, G-14, G-16) reported in the literature [1]. In Figure 7b,c, there exist larger *R*_h_ and smaller *R*_h_, which means that vesicles coexist with small micelles [61]. In addition, there are three peaks in which *R*_h_ is smaller than 1 nm for 4C_14_SAZ and C_16_SAZ. These peaks are not assigned to micelles and may be induced by the unique structure of oligomeric surfactants with multipolar head groups [62,63].

### 2.7. Antibacterial Performance

**Figure 8:**
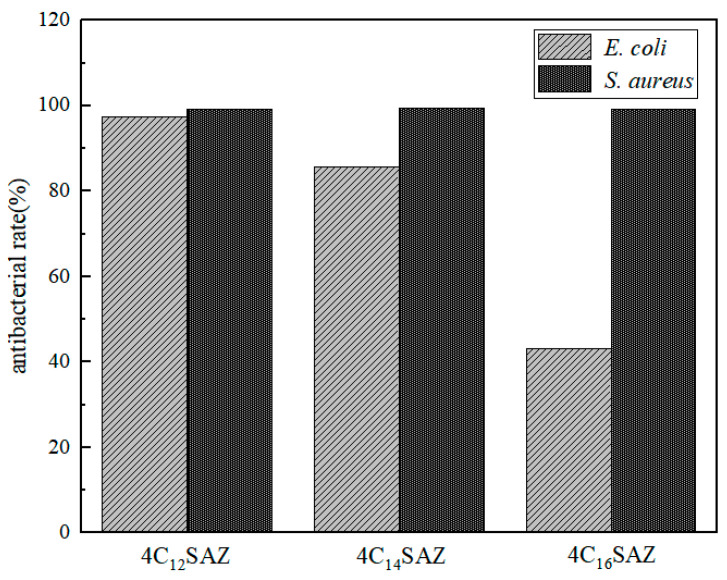
The antibacterial rates of synthesized 4C_n_SAZs against bacteria (*E. coli.* and *S. aureus*).

The antibacterial activities of 4C_n_SAZs were tested against two different microorganisms (*E. coli* and *S. aureus*) at 4C_n_SAZs concentration of 0.5 g/L, and the evaluation results are shown in Table 4 and Figure 8. As can be seen from Table 4, 4C_n_SAZs exhibit good antimicrobial activities, and this is attributable to the fact that there are four N^+^ groups and four alkyl chains in the zwitterionic tetrameric surfactants that may facilitate more electrostatic and hydrophobic interactions with the cell membrane, which increases the permeability of the cell membranes and leads to the leakage of cell contents and the disturbance metabolisms of microorganisms [64]. The antibacterial rates decrease with the increase of alkyl chain length, suggesting that there exists a distinct relationship between structure and antimicrobial activities for 4C_n_SAZs. The optimum length of the alkyl chain for 4C_n_SAZs is 12. That is, 4C_12_SAZ has the best antimicrobial activity among 4C_n_SAZs. The increasing of the alkyl chain length of 4C_n_SAZs will strengthen their hydrophobic character and decrease water solubility, which may be also supported by the lower CAC for surfactants with longer alkyl. These test results are in accordance with the antibacterial effect of other oligomeric surfactants reported in the literature [48].

Although it is wished that a compound has similar bactericidal effect on Gram-positive and Gram-negative bacteria, it is not always the case for different bacterial strains. As shown in Table 4 and Figure 8, 4C_n_SAZs show higher activities against Gram-positive bacteria (*S. aureus*) than Gram-negative bacteria (*E. coli*), which is due to the cell wall of *S. aureus* that is more permeable and disturbed than *E. coli*. According to literature data [65,66], the cell wall of *S. aureus* is very simple, and mainly composed of a mesh-like structure, while *E. coli* has a more complex cell wall that contains a layer of peptidoglycan between the cytoplasmic membrane and an outer membrane that includes lipopolysaccharides cross-linked by divalent cations. The above complex structure helps to maintain the outer membrane stable and makes lipophilic molecules more difficult in permeating into cell wall.

### 2.8. Emulsification Performance

Emulsification is one of the most important surface properties of surfactants at the application field, and emulsion stability is mainly dependent on the ability of surfactants to reduce surface tension and the mechanical strength of the interface film formed in the emulsion [67].

**Figure 9:**
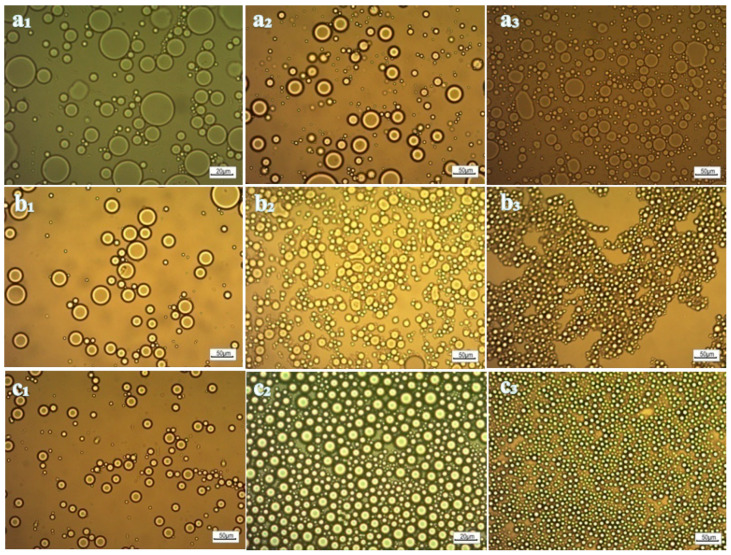
Microscopic photos of 4C_n_SAZs emulsion by emulsified kerosene at different concentrations: 4C_12_SAZ: (**a_1_**) 0.1 mmol L^−1^, (**a_2_**) 1 mmol L^−1^, (**a_3_**) 10 mmol L^−1^; 4C_14_SAZ: (**b_1_**) 0.1 mmol L^−1^, (**b_2_**) 1 mmol L^−1^, (**b_3_**) 10 mmol L^−1^, 4C_16_SAZ: (**c_1_**) 0.1 mmol L^−1^, (**c_2_**) 1 mmol L^−1^, (**c_3_**) 10 mmol L^−1^.

The emulsification performance of 4C_n_SAZs was studied at different concentrations at 298.15 K, and is shown in Table 5, and the microscopic morphology of the emulsion is given in Figure 9. It can be shown that the emulsification of 4C_n_SAZs is better than the conventional surfactant that has the same alkyl chain length such as CTAB and SDS. This may be because there are four alkyl chains and four polar head groups in the same molecule and the whole molecule is electrically neutral. The *A*_min_ value of the single alkyl chain in a 4C_n_SAZs molecule is less than that of *A*_min_ of the traditional single-chain surfactant molecule. This means that 4C_n_SAZs molecules arrange more tightly in the interface film, and thus enhance the mechanical strength of the interfacial film formed. The emulsion stability increases with the increase of the alkyl chain length, and this conclusion is attributed to a strong hydrophobic effect among the long alkyl chains [68].

The emulsification properties of surfactants can be reflected in the size of the emulsion droplets, and the smaller droplets and the narrower droplet size distribution demonstrate that surfactants have high emulsification capability and emulsion stability [69]. As can be seen from Figure 9, the droplet size and the droplet size distribution are smaller and narrower with the increase of the alkyl chain length and concentration for 4C_n_SAZs, respectively. In addition, from the microscopic images, it is interesting to note that the 4C_n_SAZs emulsion exists in the form of multiple emulsions. This may be caused by the following factors: First, the four alkyl chains in the same molecule are connected by chemical bonds and thus the alkyl chain space is less influenced by electrostatic repulsion than that of conventional ionic surfactants. Second, the decrease of electrostatic repulsion among different 4C_n_SAZs and electrostatic attraction between two head groups with opposite charges in the single molecule may be inclined to make alkyl chains arranged more packly in the interface [70]. Third, the 12 strong hydrophilic groups including N^+^, –OSO_3_^−^, –O– in the same molecule may retain a lot of water molecules. The above factors all favor enhancing the mechanical strength and viscosity of the interface film and reducing the formed droplet coalescence [71].

## 3. Experimental Methods and Materials

### 3.1. Materials

Pentaerythritol (analytical grade, Tianjin Zhiyuan Chemical Reagent Co., Ltd., Tianjin, China); Potassium hydroxide (analytical grade, Shanghai McLean Biochemical Technology Co., Ltd., Shanghai, China); Dimethyl sulfoxide, epichlorohydrin, dichloromethane, hydrochloric acid, anhydrous ethanol, ethyl acetate, sodium hydroxide (analytical grade, Tianjin Damao Chemical Reagent Factory, Tianjin, China); *N*,*N*-Dimethyldodecylamine, *N*,*N*-Dimethyltetradecylamine, *N*,*N*-Dimethylhexadecylamine (90%, Tixiai Chemical Industry Development Co., Ltd., Shanghai, China); Acetone (analytical grade, Tianjin Dongfang Chemical Plant, Tianjin, China), Trichloromethane (analytical grade, Shanghai Chemical Reagent Factory, Shanghai, China); Chlorosulfonic acid, Sodium dodecyl sulfate (SDS), Cetyl trimethyl ammonium bromide (CTAB) (analytical grade, Shanghai Aladdin Biochemical Technology Co., Ltd., Shanghai, China).

### 3.2. Synthesis of Zwitterionic Tetrameric Surfactants

#### 3.2.1. Synthesis of Pentaerythritol Tetraglycidyl Ether (I)

Pentaerythritol tetraglycidyl ether was prepared by a cheap etherification after open ring of ECH and alkanol under alkaline catalyst. Dimethyl sulfoxide (70 mL), pentaerythritol (0.033 mol), and potassium hydroxide (0.17 mol) were added to the 250-mL three-necked flask, and then stirred until all the solid materials were dissolved. Epichlorohydrin (0.165 mol) was slowly dropped into the flask at room temperature, and the reaction mixture was heated to 50 °C and maintained for 8 h. After reaction, the mixture was filtered, and the filter cake was washed with dichloromethane. The resulting filtrate is distilled under reduced pressure to get the pale-yellow oily liquid (the intermediate I)

#### 3.2.2. Synthesis of Quaternary Ammonium Tetrameric Surfactant (II)

This is a common quaterization with epoxy groups and alkyl dimethylamines. The intermediate I (0.02 mol) and alkyl dimethylamine (0.1 mol) were added to an ethanol solution (50 mL) containing hydrochloric acid (0.1 mol), and the reaction mixture was refluxed for 48 h. Then, ethanol in the mixture was removed by rotary evaporation to obtain a pale yellow viscous solid. The solid was recrystallized with acetone three times, followed by vacuum drying to obtain a white solid power (the intermediate II).

#### 3.2.3. Synthesis of Zwitterionic Tetrameric Surfactants (4C_n_SAZs)

**Scheme 1:**
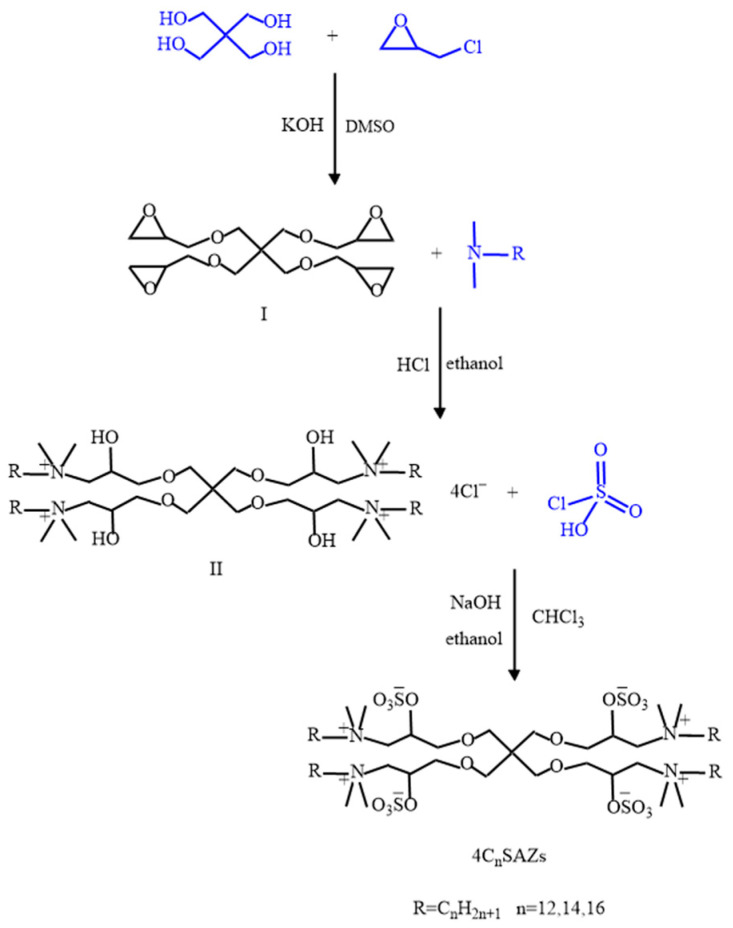
Synthesis of zwitterionic tetrameric surfactants (4C_n_SAZs, *n* = 12, 14, 16).

The synthesis of 4C_n_SAZs was carried out by mature sulfation processes of hydroxy groups. First, the intermediate II (0.01 mol) and trichloromethane (30 mL) were added to the 250-mL three-necked flask. Trichloromethane (20 mL) containing chlorosulfonic acid (0.044 mol) was dropped into the reaction flask at the ice bath under stirring. After 6 h, the reaction solution was adjusted to ca. pH 10 by sodium hydroxide alcohol solution (20 wt%). The mixed solution was filtered to remove inorganic salts, and the filtrate was distilled by rotary evaporation to remove trichloromethane and alcohol. The resulting solid was recrystallized with ethyl acetate/acetone mixed solvent to give the final compound 4C_n_SAZs (*n* = 12, 14, 16) as a white solid. The synthetic route of 4C_n_SAZs (*n* = 12, 14, 16) is given in the Scheme 1.

### 3.3. Methods

#### 3.3.1. Surface Tension Measurements

The surface tension of 4C_n_SAZs aqueous solution was measured by QBZY-2 fully automatic surface tensiometer (Shanghai Fangrui Instrument Co., Ltd., Shanghai, China) using the Wilhelmy plate method [42]. The measurements were conducted until the variations in surface tension were less than 0.2 mN m^−1^ over 5 min. The measurements were carried out at 25 °C, 35 °C, 45 °C, and 55 °C. The averages were obtained by three-run measurements.

#### 3.3.2. Steady-State Fluorescence Spectrum

Fluorescence measurements were conducted with LS-55 fluorescence spectrometer (Perkin-Elmer, Waltham, MA, USA). Under excitation, recording the spectrum between 340 nm and 440 nm with an excitation wavelength of 335 nm, where the concentration of pyrene in each solution is 1 × 10^−6^ mol L^−1^. The fluorescence intensity ratio I_1_/I_3_ of the first (373 nm) and third (384 nm) vibration peaks depends on the environment of the pyrene molecule. When the ratio of I_1_/I_3_ decreases, the environment of pyrene becomes more hydrophobic [63].

#### 3.3.3. Dynamic Light Scattering (DLS)

DLS measurements were performed with a spectrometer (Zetasizer Nano ZS90, Malvern Instruments Ltd., Malvern, UK) to study the aggregation behaviors of 4C_n_SAZs molecules in aqueous solution. The scattering angle is 90°, and 4C_n_SAZs solution was filtered through a 0.45 mm membrane filter to remove any interfering dust particles [72]. All measurements were carried out three times at room temperature.

#### 3.3.4. Antibacterial Activities

The plate counting method is a common antibacterial evaluation method that can clearly reflect the antibacterial performance of a compound from a quantitative perspective. The smaller the number of growing colonies, the better the antibacterial effect [73].

The antibacterial activities of 4C_n_SAZs against two different bacteria: Gram-positive bacterium (*S. aureus* CMCC 26003) and Gram-negative bacterium (*E. coli* CMCC 44102) were performed by plate count method [64]. The standardized inoculum was prepared with tryptone (10 g), yeast extract (5 g), NaCl (10 g) and agar (10 g) in 1000 mL of water, and its pH value was adjusted to 7. The two bacteria were cultured in the inoculum at 37 °C until their concentration arrived at 5.0 × 10^6^ CFU/mL before the test. The needed surfactant solution was prepared by adding of 4C_n_SAZs into autoclaved water and making its concentration 5 g/L.

1.0 mL of the sterilized 4C_n_SAZs solution was added to 9 mL of the nutrient broths containing the two bacterial strains and deionized water, respectively 0.5 mL of the above three solutions was applied to the eight culture dishes containing the standardized inoculum and then were incubated for 24 h at 37 °C. The number of surviving bacterial colonies was counted and the antibacterial rate was calculated in terms of Formula (13). All the experiments were tested three times, and the glassware used in the experiments was sterilized in an autoclave.

(13) $$\eta =\frac{A-B}{A}\times 100\%$$

Which, η is the antibacterial rate, *A* is the number of the bacterial colony in the contrast group, and *B* is the number of bacterial colonies in the experiment group.

#### 3.3.5. Emulsion Stability Measurements

The emulsification ability of 4C_n_SAZs was assessed by the water-separation-time method [69]. First, 20 mL of 4C_n_SAZs (0.5 wt%) aqueous solution was added to the stoppered graduated cylinder, and then the same volume of oil phase (n-hexane, kerosene, and liquid paraffin). The cylinder was vigorously shaken up and down 5 times, and then stood for 1 min. The above steps were repeated five times, and the time to separate 10 mL water was recorded. The measurements were made at room temperature, and the average values were recorded three times.

The polarizing microscope (ZEISS Axio Lab.A1) was used to more truly present the emulsion state. 20 mL of kerosene and 80 mL of 4C_n_SAZs (0.5 wt%) aqueous solution was blended with the digital display high-speed dispersing homogenizer (FJ200-SH, Shanghai Huxi Industry Co., Ltd., Shanghai, China) at a speed of 3000 rpm for 20 min. The appearance of the prepared emulsion was captured to determine the degree of emulsification [74].

## 4. Conclusions

A series of zwitterionic tetrameric surfactants (4C_n_SAZs) have been synthesized by a simple and convenient method with commercially available raw materials. 4C_n_SAZs have better surface activities such as low CAC and *γ*_CAC_ in comparison with conventional surfactants. 4C_n_SAZs have four alkyl chains closely connected in the single molecule, and the whole molecule is electrically neutral. This structure characteristic of 4C_n_SAZs can enhance the hydrophobic association among alkyl chains of the same or different molecules and decrease intermolecular electrostatic repulsion, which can result in 4C_n_SAZs molecules more compact arrangement at air-water interface. The thermodynamic parameters obtained from surface tension measurements at different temperatures show that both the processes of adsorption and micellization of 4C_n_SAZs are spontaneous and that the micellization processes of 4C_n_SAZs are entropy driven. The aggregate behavior of 4C_n_SAZs shows that the alkyl chain length has a greater sensitivity for aggregate growth. The calculated values of critical packing parameters for 4C_n_SAZs can be supported by the DLS measurements. 4C_n_SAZs have good emulsification performance, and it is interesting to note that the prepared emulsions appear to exit in the form of multiple emulsions. In addition, 4C_n_SAZs have good antibacterial activities against *E. coli* and *S. aureus*, which are also consistent with the conventional zwitterionic surfactants.

## Figures and Tables

**Table 1:** Physicochemical parameters of the different surfactants.

Surfactants	Temp. (°C)	CAC (mmol L^−1^)	*γ*_CAC_ (mN m^−1^)	*Γ*_max_ (10^10^ mol cm^−2^)	*A*_min_ (nm^2^)	*π*_CAC_ (mN m^−1^)	*p*C_20_
4C_12_SAZ	25	0.236	26.12	3.64	0.46	45.84	4.87
	35	0.131	24.60	3.43	0.48	45.79	5.16
	45	0.088	23.26	3.03	0.55	45.47	5.43
	55	0.078	22.04	2.57	0.65	45.03	5.66
4C_14_SAZ	25	0.073	23.91	4.18	0.40	48.06	5.32
	35	0.042	23.21	3.90	0.43	47.17	5.56
	45	0.029	21.89	3.51	0.47	46.85	5.80
	55	0.024	20.95	3.24	0.51	46.11	5.91
4C_16_SAZ	25	0.013	22.95	5.44	0.31	49.02	5.83
	35	0.007	21.32	4.63	0.36	49.06	6.25
	45	0.004	20.83	4.24	0.39	47.91	6.48
	55	0.003	20.08	3.73	0.45	46.99	6.64
SDS	25	7.25 ^a^	34.80 ^a^	3.60 ^a^	0.48 ^b^	37.17 ^a^	2.50 ^a^
DTAB	25	15.7 ^b^	38.90 ^b^	3.17 ^b^	0.46 ^a^	33.20 ^b^	2.18 ^b^

^a^ From the literature [38,39], ^b^ From the literature [40,41].

**Table 2:** Thermodynamic parameters in the micellization and adsorption processes for 4C_n_SAZs.

Surfactants	Temp. (K)	Δ*G*^o^_mic_ (KJ mol^−1^)	Δ*H*^o^_mic_ (KJ mol^−1^)	Δ*S*^o^_mic_ (KJ mol^−1^ K^−1^)	−*T*Δ*S*^o^_mic_ (KJ mol^−1^)	Δ*G*^o^_ads_ (KJ mol^−1^)
4C_12_SAZ	298.15	−30.66	27.42	0.19	−58.08	−43.26
	308.15	−33.18	29.29	0.20	−62.47	−46.53
	318.15	−35.31	31.22	0.21	−66.53	−50.30
	328.15	−36.75	33.21	0.21	−69.97	−54.30
4C_14_SAZ	298.15	−33.57	27.71	0.21	−61.29	−45.08
	308.15	−36.11	29.61	0.21	−65.71	−48.22
	318.15	−38.28	31.56	0.22	−69.84	−51.62
	328.15	−40.02	33.57	0.22	−73.59	−54.23
4C_16_SAZ	298.15	−37.89	33.85	0.24	−71.74	−46.91
	308.15	−40.88	36.16	0.25	−77.04	−51.47
	318.15	−43.50	38.54	0.26	−82.04	−54.79
	328.15	−45.42	41.00	0.26	−86.43	−58.02

**Table 3:** The Critical arrangement parameters of 4C_n_SAZs.

Surfactants	*a*_0_ (nm^2^)	*l*_0_ (nm)	*V*_hydrophobic_ (nm^3^)	*p*	*R*
4C_12_SAZ	0.46	1.67	0.35	0.46	3.05
4C_14_SAZ	0.40	1.93	0.40	0.53	4.04
4C_16_SAZ	0.31	2.18	0.46	0.68	5.91

**Table 4:** Antibacterial activities of synthesized 4C_n_SAZs at the concentration of 0.5 g/L against bacteria (*E. coli* and *S. aureus*) by plate count method.

	Control	4C_12_SAZ	4C_14_SAZ	4C_16_SAZ
*E. coli*	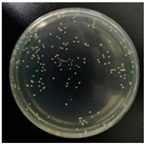	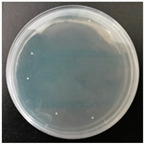	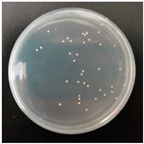	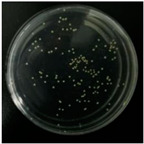
*S. aureus*	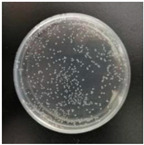	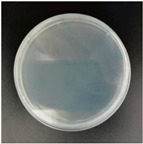	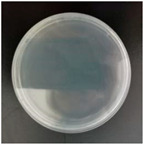	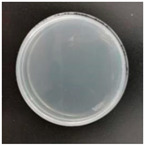

**Table 5:** Emulsification stability of the 4C_n_SAZs at room temperature.

Surfactants	Liquid Paraffin (s)	N-Hexane (s)	Kerosene (s)
4C_12_SAZ	683	934	1012
4C_14_SAZ	741	1303	2198
4C_16_SAZ	794	3247	4404
CTAB	461	626	768
SDS	347	540	712

## Data Availability

Data is contained within the article.
